# A fatal threat: *Pneumocystis jirovecii* pneumonia with rapidly progressive interstitial lung disease in anti-MDA5-positive dermatomyositis—a case report

**DOI:** 10.3389/fmed.2026.1977161

**Published:** 2026-10-30

**Authors:** Xiaolong Li, Shuhao Xu, Yinhe Feng, Rongli Wang, Xin Wang

**Affiliations:** 1 Pulmonary and Critical Care Medicine, Deyang People’s Hospital, Deyang, Sichuan, China; 2 Department of Stomatology, Deyang People’s Hospital, Deyang, Sichuan, China; 3 Pulmonary and Critical Care Medicine, The Affiliated Hospital of Southwest Medical University, Luzhou, Sichuan, China

**Keywords:** case report, melanoma differentiation-associated gene 5-positive+ dermatomyositis, metagenomic next-generation sequencing, *Pneumocystis jirovecii* pneumonia, rapidly progressive interstitial lung disease

## Abstract

*Pneumocystis jirovecii pneumonia* (PJP) is a respiratory infection caused by *Pneumocystis* organisms, representing an opportunistic fungal infection. The clinical occurrence of PJP combined with rapidly progressive interstitial lung disease (RP-ILD) in dermatomyositis (DM) have also been reported occasionally, but patients with this combination of conditions experience rapid disease progression and high mortality rates. We reported a 70-year-old female patient with dyspnea and fever, detailing her laboratory tests, computed tomography (CT) findings and bronchoalveolar lavage fluid findings. The patient's main clinical manifestations were dyspnea and fever. CT scans revealed scattered reticular opacities and ground-glass opacities in both lungs and metagenomic next-generation sequencing (mNGS) of the bronchoalveolar lavage fluid detected *Pneumocystis* jirovecii. Laboratory tests revealed positive anti-MDA5 antibodies during hospitalization. The patient was treated with high-flow oxygen therapy and methylprednisolone upon admission. Subsequently, the patient received mechanical ventilator support and was administered trimethoprim-sulfamethoxazole (TMP-SMX), plasma exchanges and immunoglobulin treatment. The patient's fever subsided, but respiratory failure and interstitial lung disease continued to progressively worsen. Ground-glass opacities, mixed ground-glass opacities, consolidations and reticular opacities were significantly more pronounced than on admission, indicating RP-ILD changes post-treatment. The patient succumbed after 13 days of treatment. PJP complicated by rapidly progressive interstitial lung disease can be fatal in patients, emphasizing the critical importance of early identification of PJP and prompt initiation of anti-PJP treatment. It is crucial to recognize that some MDA5+ DM patients may not present with the typical rash, and in certain cases, RP-ILD may be the primary manifestation. Therefore, early screening for anti-MDA5 antibodies is essential for patients presenting with unexplained early-stage RP-ILD.

## 1 Introduction

PJP is a respiratory infection caused by *Pneumocystis* organisms, representing an opportunistic fungal infection that typically occurs in individuals with compromised immune systems or immune disorders (1). Rapidly progressive interstitial lung disease is a key clinical feature of anti-melanoma differentiation-associated gene 5-positive dermatomyositis (MDA5+ DM) and is the leading cause of death in these patients. Although the clinical occurrence of PJP combined with RP-ILD in MDA5+ DM is not uncommon, patients with this combination of conditions experience rapid disease progression and high mortality rates. This report describes a case of PJP combined with RP-ILD in a patient with MDA5+ DM. The case highlights the diagnostic challenge when MDA5+ DM presents without classic rash, the radiological overlap between PJP and RP-ILD, and the therapeutic conflict between immunosuppression for RP-ILD and control of active opportunistic infection.

## 2 Case presentation

A 70-year-old female patient was admitted to the hospital with complaints of dyspnea and fever for two days. The patient did not report muscle weakness, myalgia, or muscle tenderness. The patient had a history of hypertension for 13 years (oral amlodipine, 2.5 mg once daily). On physical examination, the patient had reduced breath sounds in both lungs, a heart rate of 124 beats per minute, a respiratory rate of 24 breaths per minute, and oxygen in fingertip saturation of 85%. Bilateral basal fine crackles were audible on pulmonary auscultation. No proximal muscle weakness was observed on physical examination. Laboratory tests on the day of admission revealed the following results: white blood cell count, 12.48 × 10^^9^/L; neutrophil count, 7.78 × 10^^9^/L; lymphocyte count, 0.25 × 10^^9^/L; hemoglobin, 86 g/L; platelet count, 110 × 10^^9^/L; high-sensitivity C-reactive protein (hs-CRP), 240.80 mg/L; alanine aminotransferase (ALT), 90 U/L; aspartate aminotransferase (AST), 51 U/L; albumin, 26.8 g/L; B-type natriuretic peptide (BNP), 91.1 pg/mL. Arterial blood gas analysis showed a pH of 7.40, partial pressure of carbon dioxide (PaCO_2_) of 24.70 mmHg, partial pressure of oxygen (PaO_2_) of 57.00 mmHg, and lactic acid of 3.8 mmol/L. oxygenation index (PaO_2_/FiO_2)_ of 196 mmHg. Additionally interleukin-6 (IL-6) was 188 pg/mL, D-dimer was 0.12 mg/L,and renal function was within normal limits. Blood cultures were negative, Sputum bacterial culture showed no growth, The autoimmune antibody panel returned negative results, and vasculitis-associated markers were within normal limits.

CT revealed the presence of ground-glass opacities, mixed ground-glass opacities, consolidations, and reticular opacities in both lungs. Notably, ground-glass and reticular opacities were particularly prominent in the upper lobes of both lungs and in the subpleural region of the right lower lobe. Interstitial pneumonia was considered a potential diagnosis, although pulmonary edema could not be ruled out (Figures 1A–F). Echocardiography showed mild tricuspid regurgitation, and a venous ultrasound scan of the lower extremities found no evidence of thrombosis.

**Figure 1:**
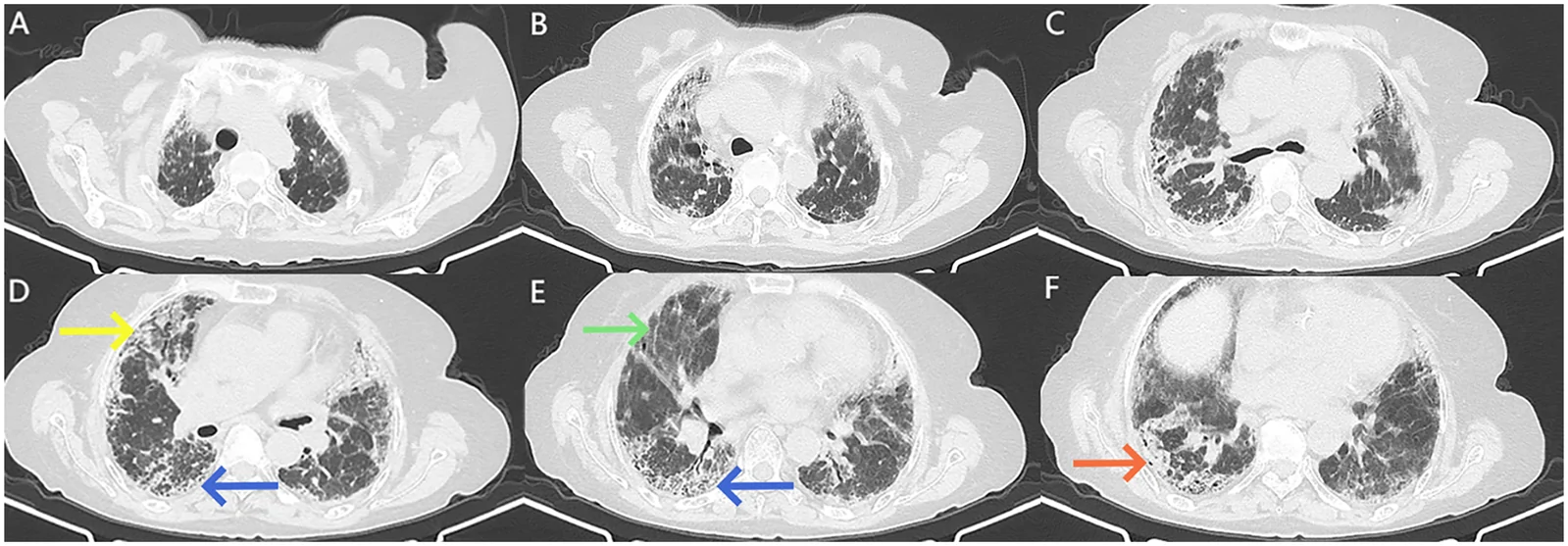
**(A–F)** computed tomography revealed the presence of ground-glass opacities (green arrow), mixed ground-glass opacities(yellow arrow), consolidations(orange arrow), and reticular opacities(blue arrow) in both lungs. Notably, ground-glass and reticular opacities were particularly prominent in the upper lobes of both lungs and in the subpleural region of the right lower lobe.

The initial diagnoses upon admission included respiratory failure, severe pneumonia, interstitial pneumonia, anemia, and hypoproteinemia. Due to severe dyspnea, high-flow oxygen therapy was initiated immediately. The patient was treated with piperacillin/tazobactam (4.5 g IV, every 8 h). moxifloxacin (0.4 g IV daily), and intravenous methylprednisolone (40 mg IV daily) to address fever, significantly elevated hs-CRP, pulmonary inflammation, and the potential for interstitial lung disease. To prevent thrombotic events, the patient was administered low-molecular-weight heparin at a dose of 6000 IU subcutaneously once daily during hospitalization. However, after two days of treatment, the patient continued to experience significant dyspnea and progressive worsening of respiratory failure. As a result, endotracheal intubation was performed, and the patient was transferred to the Respiratory Intensive Care Unit (RICU) for mechanical ventilator support.

During hospitalization, enzyme-linked immunosorbent assay (ELISA) testing for anti-MDA5 antibodies returned a positive result, with a quantitative value of 185.5 AU against a positive threshold of 32 AU. This finding led to the diagnosis of MDA5+ DM. The following additional investigations were performed on the patient: the erythrocyte sedimentation rate (ESR) was 63 mm/h, peripheral blood lymphocyte subset analysis revealed a CD4+ T-cell count of 158/*μ*L and a CD8+ T-cell count of 43/μL. The lactate dehydrogenase (LDH) level was 548 U/L, Krebs von den Lungen 6 (KL-6) was 798 U/mL, and the serum ferritin was 1650 ng/mL. Partial pressure of oxygen (PaO2) of 79.00 mmHg, oxygenation index (PaO2/FiO2) of 158 mmHg. Consequently, the treatment regimen was adjusted to include intravenous methylprednisolone (120 mg once daily) and trimethoprim-sulfamethoxazole (TMP-SMX; 80 mg trimethoprim and 400 mg sulfamethoxazole per tablet), two tablets once daily for prophylactic purposes. Additionally, the patient received intravenous immunoglobulin (20 g/day) for 5 consecutive days. Despite these treatments, the patient remained febrile and showed no significant improvement in respiratory failure. Accordingly, further laboratory investigations were performed. Repeat blood cultures were negative. The serum (1,3)-β-D-glucan level was elevated at 185 pg/mL, while the galactomannan assay was negative. Additionally, serological testing for IgM antibodies against *Mycoplasma pneumoniae*, respiratory syncytial virus, adenovirus, coxsackievirus B, rubella virus, and varicella-zoster virus all returned negative results. To investigate the cause of persistent fever and progressive respiratory failure，a bedside bronchoscopy with bronchoalveolar lavage was performed, and metagenomic next-generation sequencing (mNGS) of the lavage fluid detected *Pneumocystis jirovecii* (sequence number 26,873), with no other pathogens identified. As a result, the treatment was adjusted to include trimethoprim-sulfamethoxazole (TMP-SMX, 15 mg/kg/day), administered via nasal feeding every 6 h.

## 3 Follow-up

Following treatment, the patient's fever subsided, but respiratory failure continued to progressively worsen. To assess the lung condition, a follow-up CT scan was performed. Ground-glass opacities, mixed ground-glass opacities, consolidations and reticular opacities were seen on the latest CT, which were markedly increased compared to the initial admission (Figures 2A–F). The follow-up CT scan revealed to a diagnosis of RP-ILD. Repeat analysis of peripheral blood lymphocyte subsets demonstrated the following results: CD4+ T-cell count, 128 cells/μL; CD8+ T-cell count, 40 cells/μL. LDH was elevated at 798 U/L, serum ferritin exceeded 2000ng/mL, and KL-6 was 1090 U/mL. Arterial blood gas analysis revealed a partial pressure of oxygen of 65 mmHg and an oxygenation index of 118 mmHg. As a result, the patient underwent two plasma exchanges during the hospital stay. During hospitalization, in light of the refractory respiratory condition, ECMO support was recommended to the patient. However, the patient's family declined this treatment option due to financial constraints. Unfortunately, despite aggressive treatment, the patient was unable to be weaned from the ventilator and passed away after 13 days of treatment (Figure 3).

**Figure 2:**
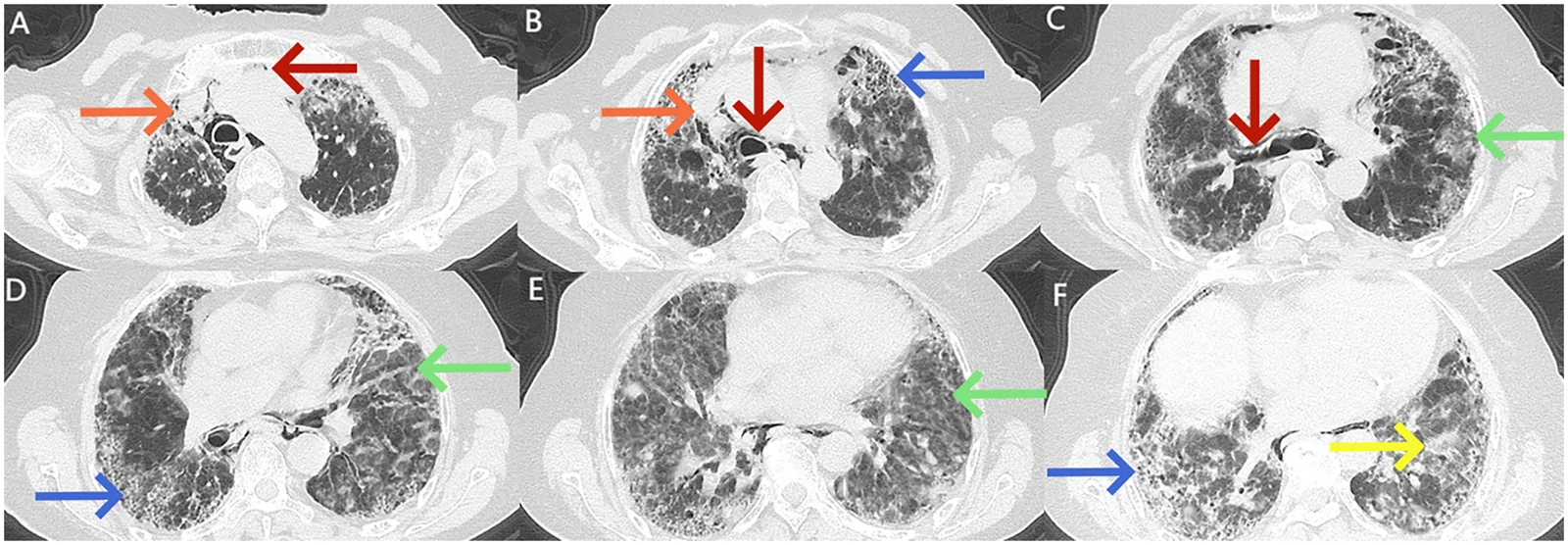
**(A–F)** follow-up CT imaging revealed consolidation in the right upper lobe (orange arrow), along with a marked increase in ground-glass opacities (green arrow) and reticular opacities (blue arrow) in the lower lobes of both lungs. In addition, a small amount of pneumomediastinum was observed (red arrow).

**Figure 3:**
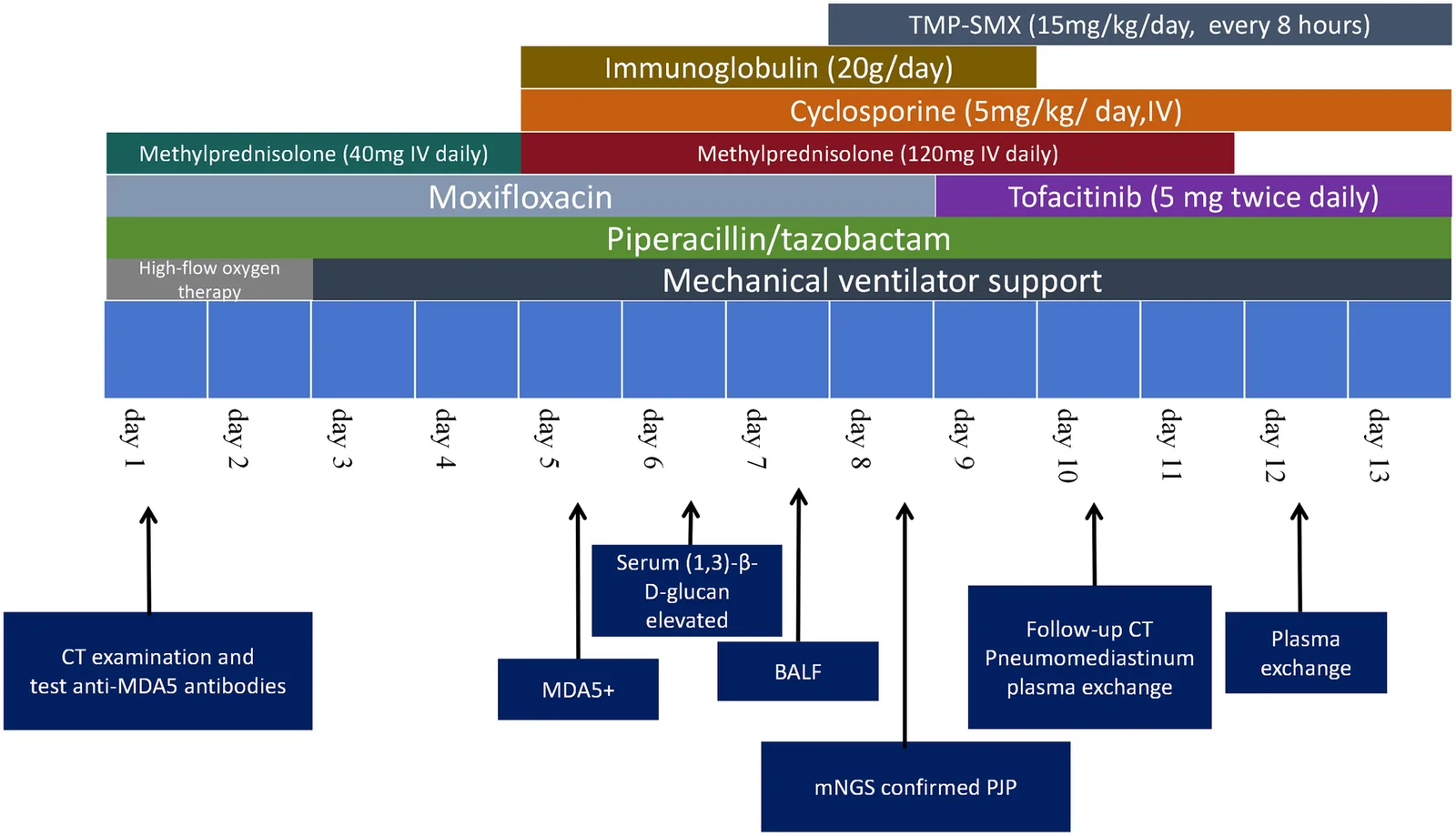
Timeline of clinical course.

## 4 Discussion

PJP is an opportunistic fungal infection that has traditionally been associated with individuals infected with human immunodeficiency virus (HIV). However, with the advent of metagenomics next-generation sequencing in recent years, more PJP cases are being diagnosed, leading to an increased understanding of the disease among physicians (2, 3). In addition to HIV infection, PJP is also commonly diagnosed in organ transplant recipients, cancer patients, and those undergoing radiotherapy or chemotherapy. Furthermore, it has been reported that individuals with abnormal immune cell counts or function—particularly those with low CD4+ T lymphocyte counts—or hypogammaglobulinemia are also at heightened risk for developing PJP (4).

MDA5+ DM is a subset of connective tissue diseases characterized by distinct skin ulcers and RP-ILD. Among various connective tissue disorders, including, systemic lupus erythematosus (SLE), rheumatoid arthritis, and scleroderma, patients with PM/DM have been reported to have the highest risk of opportunistic infections among major systemic rheumatic diseases (5, 6). Research shows that individuals with MDA5+ DM are particularly prone to developing PJP within the first three months of illness, with mortality rates higher than those in patients with other rheumatic conditions (7, 8). Factors such as advanced age, delayed initiation of anti-PJP therapy, and the need for mechanical ventilation have been identified as independent risk factors for PJP-related mortality (9, 10). Upon admission, the patient exhibited a significant reduction in peripheral blood lymphocytes, particularly CD4+ T lymphocytes- a key feature of MDA5+ DM that correlates closely with disease severity. Studies suggest that in dermatomyositis patients, elevated expression of RIG-I in T lymphocytes may trigger apoptosis, leading to reduced levels of T lymphocytes in the peripheral blood (11). Studies have indicated that severe lymphopenia, particularly a reduced CD4+ T lymphocyte count, is an independent risk factor for the development of PJP in patients with MDA5+ DM. CD4+ T lymphocytes, including Th1, Th2, Th17, and Treg subsets, play a critical role in adaptive immunity within the lungs. A decrease in CD4+ T cell count reflects a state of immunosuppression. Notably, MDA5+ DM patients with a CD4+ T lymphocyte count below 200ount reμL require heightened vigilance for the onset of PJP (5, 12). Serum immunoglobulin, creatine kinase (CK) and aldolase levels were not measured at admission or during hospitalization; therefore, hypogammaglobulinemia could not be evaluated as a risk factor, which represents a limitation of the present case.

Corticosteroid use has long been recognized as a major risk factor for the development of PJP. Studies have indicated that the increased incidence of PJP may be associated with the widespread clinical application of corticosteroids, which are among the most commonly prescribed immunomodulatory agents. The underlying mechanism is primarily related to the ability of corticosteroids to suppress the proliferation and function of T lymphocytes, thereby impairing cell-mediated immune responses. Clearance of Pneumocystis jirovecii predominantly relies on cellular immunity, particularly CD4+ T lymphocytes. In an immunocompromised state, the organism can proliferate extensively in the lungs, leading to PJP (13–15). Moreover, studies have shown that patients receiving concurrent corticosteroid and immunosuppressive therapy are at a relatively higher risk of developing PJP. Additionally, it is noteworthy that a small proportion of patients treated with immunosuppressants alone, without corticosteroids, also remain at risk for PJP (16–18).

In this case, the patient was initiated on high-dose corticosteroids along with prophylactic TMP-SMX against PJP upon diagnosis of MDA5+ DM. Clinical studies have consistently demonstrated that prophylactic administration of TMP-SMX in MDA5+ DM patients significantly reduces the incidence of PJP and may lead to improved clinical outcomes (5, 8, 16). However, when MDA5+ DM patients develop concurrent PJP and RP-ILD, the mortality rate within the first three months is markedly elevated (19).

The diagnosis of PJP relies on a combination of symptoms, risk factors, imaging findings, and microbiology evidence (20). In this case, the patient was elderly and presented with progressive dyspnea, positive anti-MDA5 antibodies, ground- glass opacities and mixed ground-glass opacities on high-resolution computed tomography (HRCT), significantly elevated serum (1-3)-beta-D-glucan levels, and the detection of *Pneumocystis jirovecii* in bronchoalveolar lavage fluid(BALF) via mNGS. Therefore, The diagnosis of PJP was unequivocal.

RP-ILD is a significant risk factor for mortality in patients with dermatomyositis and is a major concern for clinicians due to its high fatality rate. Studies have shown that patients with MDA5+ DM 20 times more likely to develop RP-ILD compared to those who are MDA5-negative, with over 50% of MDA5+ DM patients experiencing RP-ILD during the course of their illness (21, 22). Moreover, RP-ILD has been established as a predictor of mortality in patients with MDA5+ DM complicated by PJP (19). In clinical practice, characteristic rashs such as Gottron's sign and Heliotrope sign, are commonly observed in dermatomyositis. However,in this case, the patient did not exhibit these typical rashes upon admission, with RP-ILD being the most prominent clinical manifestation.

At admission, PJP was not initially considered the leading diagnosis because the patient had no known HIV infection, no prior immunosuppressive therapy, and negative autoimmune antibodies. However, the marked lymphopenia and rapid respiratory deterioration should have prompted earlier evaluation for PJP. Serum *β*-D-glucan was not measured at admission, and BAL was performed only after progressive deterioration. Earlier *β*-D-glucan testing and BAL might have led to earlier diagnosis and therapeutic-dose TMP-SMX. This potential delay in diagnosis and treatment is an important limitation of this case.

The specific mechanism underlying RP-ILD in MDA5+ DM remains unclear, but it may be linked to the activation of transforming growth factor-*β* (TGF-*β*) related pathways (23). Additionally, studies have shown that lactate dehydrogenase (LDH) level in MDA5+ DM patients with RP-ILD are significantly higher than in those without RP-ILD, suggesting that elevated LDH is an independent risk factor for RP-ILD in these patients (24). MDA5+ DM and RP-ILD exhibit significantly higher LDH levels compared to those with chronic ILD in DM (25). Furthermore, elevated LDH in MDA5+ DM patients correlates with a higher prevalence of pulmonary dysfunction relative to MDA5- DM-ILD patients. A separate cohort study demonstrated that survivors of MDA5+ DM-ILD had significantly lower LDH levels than non-survivors, and an LDH level exceeding 355 U/L may serve as an independent high-risk factor for poor prognosis (26). KL-6, secreted by damaged bronchial epithelial cells and regenerating type II alveolar cells, shows a positive correlation with ILD severity. Serum KL-6 levels are significantly elevated in MDA5+ DM-ILD patients compared to both MDA5-negative DM-ILD patients and healthy controls. Moreover, KL-6 levels correlate positively with fibrosis scores on HRCT (27). In this case, the persistent elevation of both LDH and KL-6 levels suggests an unfavorable clinical outcome.

Although PJP and anti-MDA5-associated RP-ILD may both present with fever, hypoxemia, ground-glass opacities, and elevated LDH, their relative contributions in this patient cannot be definitively separated. At admission, the patient already had fever, severe hypoxemia, marked lymphopenia, and bilateral pulmonary abnormalities, suggesting that PJP may have been present before substantial immunosuppression. The positive anti-MDA5 antibody, elevated *β*-D-glucan, and BALF mNGS detection of *P. jirovecii* confirmed coexisting PJP and MDA5+ DM. The subsequent rapid progression of ground-glass opacities, consolidation, and reticular opacities despite anti-PJP therapy indicates that RP-ILD was the predominant driver of terminal respiratory failure, while PJP contributed to the initial severity and therapeutic dilemma. Elevated LDH is a common feature of PJP and may provide a diagnostic clue for PJP; however, it is not a specific indicator for PJP. Therefore, a comprehensive diagnosis should be established by integrating clinical manifestations, imaging findings (e.g., ground-glass opacities), and pathogen detection (e.g., bronchoalveolar lavage fluid testing). LDH and KL-6 are non-specific biomarkers and should not be used alone to differentiate PJP from RP-ILD (13, 28).

Methylprednisolone 40 mg/day was started on admission because of severe hypoxemia, fever, markedly elevated hs-CRP, and suspected interstitial pneumonia. Broad-spectrum antibiotics were also initiated because severe infection was suspected. We acknowledge that initiating corticosteroids before excluding infection, including PJP, is controversial. In retrospect, earlier PJP evaluation should have been performed. This represents an important limitation of the initial management.

Following confirmation of anti-MDA5 antibody positivity, the methylprednisolone dose was increased to 120 mg/day, and intravenous immunoglobulin and plasma exchange were administered. Corticosteroid pulse therapy, tacrolimus, cyclophosphamide, and rituximab were not administered because of the patient's advanced age, severe lymphopenia, liver injury, suspected PJP, and the high risk associated with further immunosuppression. However, current recommendations for anti-MDA5-associated RP-ILD generally include early combined immunosuppression with high-dose corticosteroids and immunosuppressants. Although the treatment regimen was individualized, it may still have been insufficient. This case highlights the difficult therapeutic dilemma of treating PJP while simultaneously controlling potentially fatal inflammatory interstitial lung disease.

Furthermore, research has confirmed that a reduction in lymphocyte count is also a key factor contributing to mortality in RP-ILD (29). Currently, the diagnosis of RP-ILD relies on HRCT, which typically reveals interstitial changes (such as ground-glass opacities, reticular opacities) over a short period of time. In our case, HRCT showed rapidly progressive ground-glass opacities and interstitial changes within just 10 days. In this case, follow-up CT revealed the presence of mild pneumomediastinum in the patient. Previous studies have reported that pneumomediastinum is one of the complications associated with MDA5+ DM, with an estimated incidence ranging from 13% to 37%. Pneumomediastinum predominantly occurs in severe cases complicated by RP-ILD. The primary mechanism is thought to be related to elevated intra-alveolar pressure leading to alveolar rupture, thereby allowing air to escape into the mediastinum (30, 31), cause bronchial wall injury, and corticosteroid therapy may impair alveolar integrity, contributing to the development of pneumomediastinum. Furthermore, The occurrence of pneumomediastinum may, to some extent, reflect the severity of the underlying disease (32, 33).

For the treatment of PJP, TMP-SMX is the first-line drug of choice. In this patient, prophylactic TMP-SMX was initiated concurrently with high-dose methylprednisolone. Following the diagnosis of PJP by mNGS of bronchoalveolar lavage fluid, the patient was transitioned to therapeutic-dose TMP-SMX. Research indicates that PJP patients with a PaO_2_＜70 mmHg or Alveolar-arterial Oxygen Difference (A-aDO_2_) gradient≥35 mmHg may benefit from glucocorticoid treatment (34). The mortality rate exceeds 80% when RP-ILD occurs in MDA5+ DM patients (35).

As is well-established, anti-MDA5 antibody plays a critical role in the pathogenesis and progression of the disease. Therefore, therapeutic plasma exchange was administered during the patient's hospitalization. This intervention was primarily selected for its capacity to directly remove anti-MDA5 antibodies from the serum, as well as to effectively clear inflammatory cytokines—including interleukin-6 (IL-6) and tumor necrosis factor-alpha (TNF-α)—from the circulation (36, 37).

Treatment with glucocorticoids combined with plasma exchange has been shown to improve the survival rate of MDA5+ DM patients with RP-ILD (36). Unfortunately, despite aggressive treatment, this patient did not survive. This highlights the significant challenges clinicians face in managing PJP with RP-ILD in MDA5+ DM patients, underscoring the complexity and intractability of this condition.

Additional potent immunosuppressive agents, including calcineurin inhibitors, cyclophosphamide, and JAK inhibitors, were considered for the treatment of RP-ILD but were withheld because of active PJP and the risk of further compromising immune function. The patient received corticosteroids, intravenous immunoglobulin, and plasma exchange. This case illustrates the therapeutic dilemma between controlling RP-ILD and managing an active opportunistic infection.

In addition, plasma exchange therapy has shown a positive impact on improving survival, particularly in patients with MDA5+ DM-related refractory RP-ILD (38). In this case, two plasma exchanges were performed, but the results were unsatisfactory. A study from China confirmed that while pirfenidone has limited effectiveness in treating acute paroxysmal RP-ILD, it may contribute to better long-term outcomes in RP-ILD patients (39). Furthermore, studies have suggested that lung transplantation with extracorporeal life support is a promising option for patients with MDA5+ DM-related refractory RP-ILD (40).

## 5 Conclusion

The development of both PJP and RP-ILD in patients with MDA5+ DM is associated with high mortality (7). The co-occurrence of PJP and RP-ILD can be fatal in these patients, making early identification of PJP and prompt initiation of anti-PJP treatment is critical. Additionally, it is important to recognize that some MDA5+ DM patients may not present with the typical rash, and in certain cases, RP-ILD may be the primary manifestation. Therefore, early screening for anti-MDA5 antibodies is essential for patients presenting with unexplained early-stage RP-ILD, particularly in the absence of typical rash. This case illustrates the educational value of recognizing PJP and RP-ILD in MDA5+ DM. Clinicians should maintain a high index of suspicion for PJP in patients with MDA5+ DM, even when typical rashes are absent. The overlap between PJP and RP-ILD on imaging and the need to balance immunosuppression with infection control are critical challenges in clinical practice.

## Data Availability

The original contributions presented in the study are included in the article/Supplementary Material, further inquiries can be directed to the corresponding author.
